# *Chlamydiaceae *infections in pig

**DOI:** 10.1186/1297-9716-42-29

**Published:** 2011-02-07

**Authors:** Katelijn Schautteet, Daisy Vanrompay

**Affiliations:** 1 Department of Molecular Biotechnology, Faculty of Bioscience Engineering, Ghent University, Coupure Links 653, B-9000 Ghent, Belgium

## Abstract

*Chlamydiaceae *are Gram-negative obligate intracellular bacteria. They are responsible for a broad range of diseases in animals and humans. In pigs, *Chlamydia suis*, *Chlamydia abortus*, *Chlamydia pecorum *and *Chlamydia psittaci *have been isolated. *Chlamydiaceae *infections in pigs are associated with different pathologies such as conjunctivitis, pneumonia, pericarditis, polyarthritis, polyserositis, pseudo-membranous or necrotizing enteritis, periparturient dysgalactiae syndrome, vaginal discharge, return to oestrus, abortion, mummification, delivery of weak piglets, increased perinatal and neonatal mortality and inferior semen quality, orchitis, epididymitis and urethritis in boars. However, *Chlamydiaceae *are still considered as non-important pathogens because reports of porcine chlamydiosis are rare. Furthermore, *Chlamydiaceae *infections are often unnoticed because tests for *Chlamydiaceae *are not routinely performed in all veterinary diagnostic laboratories and *Chlamydiaceae *are often found in association with other pathogens, which are sometimes more easily to detect. However, recent studies have demonstrated that *Chlamydiaceae *infections in breeding sows, boars and piglets occur more often than thought and are economically important. This paper presents an overview on: the taxonomy of *Chlamydiaceae *occurring in pigs, diagnostic considerations, epidemiology and pathology of infections with *Chlamydiaceae *in pigs, public health significance and finally on prevention and treatment of *Chlamydiaceae *infections in pigs.

## 1. Introduction

*Chlamydiaceae *are Gram-negative obligate intracellular bacteria. They are responsible for a broad range of diseases in animals and humans. *Chlamydiaceae *primary replicate in mucosal epithelial cells of the conjunctivae, the respiratory, urogenital and gastrointestinal tract. They can survive and replicate in monocytes and macrophages and are characterized by distinct extracellular and intracellular forms. The infective elementary body (EB) is metabolically inactive. It reorganizes to a reticulate body (RB) after entering the host cell. These RBs are metabolically active and divide by binary fission within phagocytotic vesicles. Condensation to EBs and the subsequent lysis of the host cell completes the chlamydial replications cycle. Several studies, however, described the occurrence of alternative developmental stages consisting of abnormal sized, mostly enlarged RB-like structures called aberrant bodies (ABs) and their association with persistence of *Chlamydiaceae *[1].

*Chlamydiaceae *infections in pigs have been known to occur since 1955 when Willingan and Beamer [2] first isolated chlamydia from cases of arthritis and pericarditis in U.S. pigs. Massive outbreaks of chlamydiosis associated with bronchopneumonia or abortion in pigs kept under intensive animal production systems were reported in Eastern European countries and Russia between 1960 and 1970 [3-5]. In 1969, the first pig chlamydial strains were isolated in Western Europe from numerous Austrian pigs with polyarthritis, polyserositis, pneumonia, conjunctivitis or enteritis, from sows that aborted and from pigs with inapparent intestinal tract infection [6,7]. In the 1980s, chlamydial strains were isolated from healthy and sick German pigs [8,9]. In the 1990s, chlamydia was consistently isolated from pig herds in Nebraska and Iowa or detected in conjunctival specimens from pigs affected with conjunctivitis or keratoconjunctivitis in all phases of production [10]. Many of the nursing and nursery pigs with conjunctivitis from these and other herds had diarrhoea, and at necropsy most of the diarrheic pigs also showed pneumonia. Although known pathogens were believed to be the cause of the diarrhoea and the pneumonia, chlamydia was isolated from or detected in the intestines and lungs of affected pigs. During the 1990s, chlamydia was also often isolated and/or detected in German and Swiss pig herds and infections were associated with return to oestrus, abortion, enteric disease and asymptomatic intestinal infections [11-15].

Thus, as reported in the literature, chlamydial disease in pigs includes conjunctivitis, pneumonia and pseudomembranous or necrotizing enteritis, as demonstrated by experimental reproduction of infection in gnotobiotic pigs using clinical isolates [16-18]. In addition, *C. suis *is associated with pericarditis, polyarthritis and polyserositis in piglets [2] and numerous reproductive problems such as vaginal discharge, return to oestrus, abortion, mummification, delivery of weak piglets, increased perinatal and neonatal mortality [19] as well as orchitis, epididymitis and urethritis in boars [20]. For most of these disorders, the exact role of *Chlamydiaceae *still has to be determined. Eggeman et al. [21] noticed a correlation between the occurrence of the periparturient dysgalactiae syndrome (PDS) in sows and the number of animals being seropositive for *Chlamydiaceae*. However, evidence that *C. suis *is causing PDS is lacking.

*Chlamydiaceae *were and still are considered as non-important pathogens of pigs because tests for *Chlamydiaceae *are not normally performed at most veterinary diagnostic laboratories and *Chlamydiaceae *are often found in association with other pathogens.

Chlamydial infections in breeding sows, boars and piglets occur more often than originally thought. *Chlamydia abortus*, *Chlamydia pecorum*, *Chlamydia psittaci *and *Chlamydia suis *can infect pigs.

## 2. Taxonomy of chlamydial species occurring in pig

In 1999, Everett et al., [22] proposed a reassignment from the single genus *Chlamydia *into two genera, *Chlamydia *and *Chlamydophila*, based on clustering analyses of the 16S rRNA and 23S rRNA genes (Table 1). However, comparative genome analysis of strains mentioned in Table 2 is consistent with the conclusion that host-divergent strains of chlamydia are biologically and ecologically closely related. The previous taxonomic separation of the genus based on ribosomal sequences is neither consistent with the natural history of the organism revealed by genome comparisons, nor widely used by the chlamydia research community eight years after its introduction. Consequently, it was proposed to reunite the *Chlamydiaceae *into a single genus, *Chlamydia *[23] (Table 1). Accordingly, the chlamydia nomenclature is used here.

**Table 1:** Taxonomy of chlamydial organisms.

	*Chlamydial taxonomy before 1999*		***Chlamydial taxonomy since 1999 (Everett et al., 1999 ***[22]**)**				***Chlamydial taxonomy used in the Twenty-first century (Stephens et al., 2009 ***[23]**)**	
**Order**	**Chlamydiales**		**Chlamydiales**				**Chlamydiales**	

**Family**	***Chlamydiaceae***		***Chlamydiaceae, Simkaniaceae, Parachlamydiaceae, Waddliaceae***				***Chlamydiaceae, Simkaniaceae, Parachlamydiaceae, Waddliaceae***	

**Genus**	***Chlamydia***		***Chlamydia***		***Chlamydophila***		***Chlamydia***	

**Species**	***C. trachomatis***	Trachoma biovar	*C. trachomatis*	Trachoma biovar			*C. trachomatis*	Trachoma biovar
		LGV biovar		LGV biovar				LGV biovar
		Murine biovar	*C. muridarum*				*C. muridarum*	
		**Porcine biovar**	***C. suis***				***C. suis***	
	*C. pneumonia*	Human biovar			*Cp. pneumonia*	TWAR biovar	*C. pneumonia*	TWAR biovar
		Koala biovar				Koala biovar		Koala biovar
		Equine biovar				Equine biovar		Equine biovar
	***C. psittaci***	**Avian subtype**			***Cp. psittaci***		***C. psittaci***	
		**Abortion subtype**			***Cp. abortus***		***C. abortus***	
		Feline subtype			*Cp. felis*		*C. felis*	
		Guinea-pig subtype			*Cp. caviae*		*C. caviae*	
	***C. pecorum***				***Cp. pecorum***		***C. pecorum***	

**Table 2:** Completed *Chlamydia *genomes.

Species	Strains	Completed in	Group	Reference
*C. trachomatis*	D/UW-3/Cx	1998	Berkeley/Stanford	[79]
*C. trachomatis*	A/Har-13	2005	RML NIAID	[80]
*C. trachomatis*	LGV L2/434	2008	Sanger Institute	[81]
*C. trachomatis*	LGV L2/UCH-1	2008	Sanger Institute	[81]
*C. pneumoniae*	CWL029	1999	Berkeley/Stanford	[82]
*C. pneumoniae*	J138	2000	Yamaguchi University	[83]
*C. pneumoniae*	AR39	2000	TIGR	[84]
*C. pneumoniae*	TW-183	2007	ALTANA	[85]
*C. pneumoniae*	Koala	2008	University of Maryland	[86]
*C. muridarum*	Nigg	2000	TIGR	[84]
*C. caviae*	GPIC	2003	TIGR	[87]
*C. abortus*	S26/3	2005	Sanger Institute	[88]
*C. felis*	Fe/C-56	2006	Yamaguchi University	[89]
*C. psittaci*	6BC	2008	University of Maryland	[86]
*C. pecorum*		2008	University of Maryland	[86]
*Protochlamydia*	UWE25	2004	University of Vienna	[90]
*Simkania*		2008	University of Maryland	[49]

Adapted from Stephens et al. [23].

### 2.1. Chlamydia abortus (ruminant C. psittaci serovar 1)

*Chlamydia abortus *was formerly classified as ruminant *C. psittaci *serotype 1. This species has a distinct serotype and the ribosomal and outer membrane protein A (*ompA*) sequences are nearly 100% conserved. An extra chromosomal plasmid has not been identified in any of the *C. abortus *strains. The ruminant strain B577 (ATCC VR 656) is regarded as type reference strain.

*Chlamydia abortus *is one of the main infectious causes of abortion in sheep, cattle and goats in many countries around the world [24]. In addition, the pathogen has also been associated with abortion in horses, rabbits, guinea pigs, mice and pigs. Furthermore, pregnant women working with animals infected with *C. abortus *are at risk, as *C. abortus *is also a zoonotic agent able to cause abortion in humans [25]. *Chlamydia abortus *strains in pigs are primarily associated with abortion and weak neonates. So far, transmission of *C. abortus *from pigs to humans has not been reported.

### 2.2. Chlamydia pecorum

*Chlamydia pecorum *strains are serologically and pathogenically diverse and have been isolated only from mammals. Two strains, E58 and Koala II, have an extra chromosomal plasmid, pCp. The type strain for *C. pecorum *is E58 (ATCC VR 628).

The presence of *C. pecorum *has been established in ruminants [26], pigs and koalas (marsupials) [22]. *Chlamydia pecorum *has been associated with a wide range of diseases. In koalas, *C. pecorum *causes conjunctivitis, reproductive disease, infertility and urinary tract disease. Additionally, *C. pecorum *has been associated with urogenital tract infections, inapparent intestinal infections, abortion, conjunctivitis, mastitis, encephalomyelitis, enteritis, pneumonia, polyarthritis, pleuritis, pericarditis in sheep, goats, cattle, horses and pigs [26-28].

### 2.3. Chlamydia psittaci

*Chlamydia psittaci *primarily infects birds, but has caused sporadic zoonotic infections in humans. The bacterium is spread between birds mainly by inhalation of contaminated aerosols of ocular or nasal secretions and contaminated dust from feathers and faecal material. The transmission can also be vertical through the egg. *Chlamydia psittaci *has nine known genotypes (A-F, E/B, M56 and WC) which are all considered to be transmissible to humans. Several strains have an extra chromosomal plasmid. The type strain for *C. psittaci *is 6BC (ATCC VR 125).

In pigs, *C. psittaci ompA *genotype A has been isolated from the genital tract of a Swiss breeding sow [29]. Additionally, *C. psittaci *has been isolated from the lungs of a Belgian sow [30]. In the latter study, the genotype could not be defined as the nucleotide sequence of the cloned *C. psittaci ompA *gene (Genbank Accession No. AY327465) was found to be 99.3, 99.1 and 98.9% identical to that of *C. psittaci *CP3, 6BC and MN Zhang, respectively. A significant relationship was found between *C. psittaci *infections in pigs and keeping poultry on the farm [21,30].

### 2.4. Chlamydia suis

Before 1999, *Chlamydia suis *strains were referred to as *C. trachomatis *because of *ompA *DNA sequence homology [28]. Currently, the only known natural host of *C. suis *is the pig. Several strains have an extra chromosomal plasmid, pCs [25]. The type strain of this species, S45 (ATCC VR 1474) was isolated in Europe in the late 1960s from faeces of an asymptomatic pig in Austria [6]. This strain is tetracycline sensitive (Tc^S^), as other chlamydial species. *Chlamydia suis *strains expressing a stable tetracycline resistant phenotype associated with the presence of a resistance gene, *tet*(C), in the chromosome, have been isolated in farms in Iowa and Nebraska [31], in Italy [32] and in Belgium [33]. *Chlamydia suis *in pigs has been associated with conjunctivitis, rhinitis, pneumonia, enteritis, PDS, reproductive disorders such as return to oestrus (early embryonic dead in more than 50% of the sows) and inferior semen quality (decrease of sperm cell motility and dead of more than 50% of the sperm cells) and apparently asymptomatic infections [10,15-18,21,34-36]. A high degree of genetic diversity was observed in *C. suis *when compared to other chlamydial species [22,37]. Incongruent reports on the pathology of *Chlamydiaceae *in pigs and variations in virulence support the theory of genetic diversity [15,21,34,38,39].

## 3. Diagnostic considerations

Diagnostic laboratories do not routinely test for *Chlamydiaceae *in pigs. Cell cultures are the most convenient method for the isolation of *Chlamydiaceae*. However, with pig strains, this method has been only partially successful because it is difficult to grow the bacteria on the established transformed cell lines (HeLa and McCoy) for chlamydial culture and techniques had to be modified. Guseva et al. [40] used pig genital epithelial cells which were cultured ex vivo for dissecting the hormonal modulation of several aspects of *C. trachomatis *pathogenesis and infection. *Chlamydia suis *(S45) was used in this in vitro model. Consequently, these pig primary cells could be suitable in theory for chlamydial diagnosis in pigs. Moreover, they can be frozen at -80°C in DMEM-F-12 medium with 10% dimethyl sulfoxide (DMSO) for several weeks and they can reform monolayers in 3 to 5 days after thawing. However, continuous cell lines are still more convenient and less expensive. Schiller et al. [41] cultivated porcine *Chlamydiaceae *under various conditions. The combination of centrifugation assisted cell culture infection and cycloheximide treatment of cell coverslip cultures provided the highest inclusion numbers with all chlamydial strains. Interestingly, the use of Iscove's modified Dulbecco's medium instead of Eagle's minimal essential medium significantly increased *C. suis *inclusion counts in Vero cells. However, *C. suis *and *C. pecorum *inclusion numbers were markedly increased in CaCo cells when compared to Vero cells.

The enzyme-linked immunosorbent assay (ELISA) has been very popular for chlamydial antigen detection because it is easy to use. Kits designed to detect *C. trachomatis *in humans have been used extensively in veterinary medicine because most of them, (especially the earlier developed kits) detect the chlamydial family-specific LPS antigen and therefore will detect all chlamydial strains. However, the most important drawback of these tests are the cost and the lack of sensitivity and specificity [21,42].

Immunohistochemical staining of histological sections is often used as more veterinary diagnostic laboratories are using equipment to automate the staining. A *Chlamydiaceae *family-specific mouse monoclonal antibody directed against the chlamydial lipopolysaccharide (LPS; Clone ACI-P, Progen, Heidelberg, Germany) and a chlamydial genus-specific mouse monoclonal antibody (IgG1) directed against recombinant *C. trachomatis *HSP60 (clone A57-B9, Milan Analytica AG, La Roche, Switzerland) have been used with detection by the streptavidin-biotin method (Dako ChemMate™ detection kit; Dako Diagnostics, Heverlee, Belgium) [43]. Unfortunately, aberrant bodies are stained less, especially when using anti-HSP60 antibodies.

PCR techniques have been developed for use in veterinary medicine. They are replacing isolation for the detection of *Chlamydiaceae *in animals. If properly designed, the specificity is excellent and the sensitivity equals or exceeds well-controlled isolation procedures. For bio-safety reasons, the sample can be inactivated prior to testing. Current PCR tests for detection of *Chlamydiaceae *species occurring in pigs, target the *ompA*, the 16S-23S rRNA or the *incA *gene [22,38,44,45]. Targeting the 16S-23S genes increases sensitivity, as multiple copies of those genes are usually present in the organism. However, cross-reactions with other bacteria can interfere. The sensitivity and specificity of PCR also varies on sample preparation. Reagents designed to stabilise the DNA should be considered when a delay in processing the sample is anticipated. DNA samples can be prepared using inexpensive reagents or using commercial available kits. Sensitivity increases by targeting a relatively short DNA segment, using a nested procedure or using real-time PCR techniques. The nested PCR procedure is very sensitive. However, extreme care should be taken when manipulating the reaction in order to decrease the risk of contamination. The real-time PCR requires a labelled probe and special equipment, which increases costs. The sensitivity of this test is approximately the same as the nested PCR and contamination problems and labour are reduced as it consists of only one reaction in a closed system, often provided with uracil-N-glycosylase (UNG) to prevent post PCR carry over.

Some years ago, Sachse et al. [46] designed a microarray hybridization assay for the identification of chlamydial species using the ArrayTube™ platform (Clondiag Chip Technologies, Jena, Germany). The test proved suitable for unambiguous species identification of chlamydial cell cultures and showed potential for direct detection of these bacteria from clinical tissues. Unfortunately, routine testing for *Chlamydiaceae *in pigs is not possible because the price (24 € per sample) is not feasible.

Sequencing of PCR products can allow comparison between the sequences of reference chlamydial isolates and this information can be used in phylogenetic analysis for classification and epidemiological purposes. However, sequence analysis of the outer membrane protein A (*ompA*) gene is to our opinion not advisable for pig isolates, because of high sequence homology between the *ompA *gene of *C. psittaci *and *C. abortus *[47]. Multi locus sequence typing (MLST) has also been used for typing chlamydial species which occur in pigs. Multi locus sequence typing has been described for *C. abortus*, *C. pecorum *and *C. psittaci *[48,49].

## 4. Epidemiology of infections with *Chlamydiaceae *in pigs

### 4.1. Serology

The earliest serological data on the occurrence of chlamydial organisms in European pigs originate from 1966. At that time, Wilson and Plummer [50] discovered antibodies against *Chlamydiaceae *by capillary agglutination testing in 23% of sera derived from pigs in Great Britain.

At present, chlamydial infections are endemic in the Belgian pig population [30] as 240 (96.5%) of 249 examined fattening pig farms were seropositive by use of a recombinant ELISA detecting *Chlamydiaceae *family-specific antibodies. In this ELISA the plate was coated with the recombinant Major outer membrane protein (MOMP) of an avian *C. psittaci *genotype D strain [51]. Recombinant MOMP was prepared in COS-7 cells [52] instead of *E. coli *[53], in order to mimic eukaryotic glycosylation of MOMP and avoid *E. coli *fragments (LPS) which will be unavoidably present in purified recombinant proteins expressed in prokaryotes. In Germany, *Chlamydiaceae *seroprevalence rates in sows and boars are 33 to 72% and 10 to 47%, respectively [21]. For Switzerland, seroprevalence rates of 62% in sows, 6.9% in piglets younger than 4 weeks and 48.1% in piglets older than 4 weeks are reported [54]. These studies used an LPS-based ELISA described by Wittenbrink et al. [14]. In Italy, 63.5 to 80.3% of the finishing pigs were serologically positive when using a purified elementary body-based micro-immunofluorescence test (MIF) [55]. Bagdonas et al. [42] found a seroprevalence rate of 7.7% in Lithuanian pigs when using the complement fixation test (CFT). However, serological results obtained by using chlamydial LPS or whole elementary bodies should be handled with some reservations, as serological cross-reactions with antibodies against other pathogens do occur [56-59]. Moreover, the CFT is less sensitive and less specific than ELISA [60].

Qiu [61] and Zhou and Qiu [62], reported a seroprevalence rate in china of 11% and 80% in piglets and sows, respectively. However, most recent data originate from the Guangdong Province in Southern China revealing a seroprevalence rate of 63.38%, 41.10% and 36.25% in breeding boars, breeding sows and fattening piglets, respectively, when using a commercially marketed indirect haemaglutination assay (IHA kit; Lanzhou Veterinary Research Institute, Lanzhou, China) [63]. To our knowledge, there are no recent serological data on *Chlamydiaceae *in pigs from other countries.

### 4.2. Molecular diagnosis

Serology is useful for monitoring the *Chlamydiaceae *status in pigs, at least when using a *Chlamydia*-specific target antigen. However, all current serological tests fail to identify the causative chlamydial species. Thus, more detailed information on the prevalence of *Chlamydiaceae *in pigs originates from newly developed species-specific molecular diagnostic research.

Recently, species-specific nucleic acid amplification tests (NAATs) such as real-time PCR and microarray, detecting the ribosomal intergenic spacer and domain I of the 23S rRNA gene, have been designed [46,64]. Moreover, Dugan et al. [65] designed a PCR for detecting the tetracycline resistance (Tc^R^) gene, *tet*(C). These methods have been recently applied to examine the occurrence of different chlamydial species in pigs.

*Chlamydia psittaci *DNA was only sporadically found in pigs [29,30,33]. *Chlamydia pecorum *was also less frequently found and has been identified, using *ompA *gene sequencing, in 2% of *ompA *gene-positive boar sperm samples, in 5% of *ompA *gene-positive foetuses and in 9% of *ompA *gene-positive gut tissues [45]. *Chlamydia abortus *was identified in the lungs of a Belgian pig that accidently died after blood sampling [33]. Although, *C. abortus *is mainly linked to reproductive failure and abortions in pigs, it has previously been identified in lungs of pigs [46].

Involvement of *C. suis *was reported in the vast majority of chlamydial intestinal infections in Belgian, German and Swiss pigs. *Chlamydia abortus *was rarely found in these studies [11,12,15,29]. *Chlamydia suis *was predominantly associated with conjunctivitis in intensively kept German, Swiss and Estonian pigs [38]. Furthermore, a German study reported a high prevalence of mixed infections with *C. suis *and *C. abortus *in the lung and gut of pigs [39].

Chlamydial DNA has been discovered by nested PCR in 57.1% of the animals of a German wild boar population in Thuringia [44]. Organisms were predominantly detected in the lung. Sequencing of the amplified *ompA *segments revealed *C. psittaci, C. abortus *and *C. suis *in this wild boar population. These findings revealed a possible wildlife reservoir of these bacteria.

## 5. Pathogenicity

### 5.1. Experimental infections

The first experimental infection was performed by Pavlov et al. [66] infecting young piglets with chlamydial agents isolated from Bulgarian pigs. The infection resulted in keratoconjunctivitis, fever, anorexia and depression.

The literature describes four conjunctival *C. suis *strains: H5, H7, R22 (USA) and DC6 (Germany); 14 *C. suis *strains obtained from Italian pigs with conjunctival and/or reproductive disorders (MS1 to MS14); five intestinal strains: R19, R27, 130, 132 (USA) and S45 (Austria); and two respiratory strains R24 and R33 (USA) (Table 3). All *C. suis *strains, with the exception of S45, originated from pigs with clinical disease. Koch's postulates have been fulfilled in gnotobiotic pigs using H7 (conjunctivitis), R19 and R27 (intestinal lesions) and R33 (lung and nasal lesions) [16-18]. Strain S45 was isolated from the faeces of an asymptomatic pig, but experimental enteric infection of gnotobiotic piglets with S45 proved its pathogenic potential as it elicited significant enteric disease [67]. Interestingly, Pospischil et al. [43] reported the occurrence of aberrant *C. suis *developmental forms, indicative for a persistent infection in experimentally infected gnotobiotic pigs.

**Table 3:** *Chlamydia suis *strains isolated from pigs

		Isolated from		
		
Strain	Location	Year	Tissue	Clinical symptoms of the pig(s)
S45	Austria	1969	Intestines (faeces)	Asymptomatic infection
R19	Nebraska	1992	Intestines (faeces)	Pneumonia, enteritis, conjunctivitis
R22	Nebraska	1992	Conjunctiva	Conjunctivitis
R24	Nebraska	1992	Respiratory tract (nasal mucosa)	Upper respiratory tract disease
R27	Nebraska	1993	Intestines (colon)	Enteritis
R33	Nebraska	1994	Respiratory tract (nasal mucosa)	Pneumonia
H5	Iowa	1994	Conjunctiva	Conjunctivitis
H7	Iowa	1994	Conjunctiva	Conjunctivitis
130	Nebraska	1996	Intestines (jejenum)	Asymptomatic infection
132	Nebraska	1996	Intestines (ileum)	Asymptomatic infection
DC6	Germany	2004	Conjunctiva	Conjunctivitis
MS1	Italy	2004-2007	Conjunctiva	Conjunctivitis
MS2	Italy	2004-2007	Conjunctiva	Conjunctivitis
MS3	Italy	2004-2007	Conjunctiva	Conjunctivitis
MS4	Italy	2004-2007	Conjunctiva	Conjunctivitis and return to oestrus
MS5	Italy	2004-2007	Conjunctiva	Conjunctivitis and return to oestrus
MS6	Italy	2004-2007	Conjunctiva	Conjunctivitis and return to oestrus
MS7	Italy	2004-2007	Conjunctiva	Conjunctivitis and return to oestrus
MS8	Italy	2004-2007	Conjunctiva	Conjunctivitis and return to oestrus
MS9	Italy	2004-2007	Conjunctiva	Conjunctivitis and return to oestrus
MS10	Italy	2004-2007	Conjunctiva	Conjunctivitis and return to oestrus
MS11	Italy	2004-2007	Conjunctiva	Conjunctivitis and return to oestrus
MS12	Italy	2004-2007	Conjunctiva	Conjunctivitis
MS13	Italy	2004-2007	Conjunctiva	Conjunctivitis
MS14	Italy	2004-2007	Conjunctiva	Conjunctivitis

Experimental aerosol challenge with *C. suis *of conventional raised colostrum-fed pigs confirmed the pathogenic potential of *C. suis *for the porcine respiratory system [46,68]. In the latter studies, six week-old pigs were infected by aerosol using 10^9 ^chlamydial inclusion-forming units. All infected animals developed an acute infection characterized by a dry cough, serous nasal discharge and severe dyspnoea accompanied by wheezing, shortness of breath and breathlessness. The body temperature of the pigs rose above 40°C for at least five days post infection. Clinical signs lasted for seven days post infection.

The role of *C. suis *and *C. abortus *in reproductive problems is still controversial. One of the reasons for this is that there are often ethical objections when willing to proof Koch's postulates, as it requires *C. suis *or *C. abortus *experimental infections of *Chlamydiaceae*-negative sows, at different stages of gestation. Moreover, it is extremely difficult to find *Chlamydiaceae*-negative sows. To our knowledge, there is only one study that deals with an experimental *C. abortus *infection in sows. Experimental infections of four sows with the BS ruminant *C. psittaci *serovar 1 strain at 42 days of gestation resulted in infection of fetal membranes, but failed to induce abortion [69].

Guscetti et al. [70] studied the pathogenicity of a *C. psittaci *isolate of pigeon origin (T49/90) in three day-old gnotobiotic piglets. The animals were infected intragastrically resulting in a productive enteric infection with mild lesions, weak systemic dissemination, and faecal shedding, indicating the pig as a potential host for avian *Chlamydiaceae*. Oral administration of *C. suis *was more virulent for three day-old gnotobiotic piglets than oral inoculation of *C. psittaci *(pigeon strain T49/90) or *C. abortus *(S26/E) [70,71].

### 5.2. Studies on natural infections

The prevalence, the zoonotic risk and the economic impact of different chlamydial species occurring in pigs was difficult to determine because species-specific diagnostic tests were unavailable. Recently, chlamydial species-specific NAATs have been developed. These NAATs are currently being used to study the prevalence and the economic impact of different chlamydial species occurring in pigs. *Chlamydiaceae*, and especially, *C. suis *are widespread in pigs. Considering the high degree of genetic diversity observed in *C. suis *when compared to other chlamydial species [22,37], incongruent reports on the pathology of *Chlamydiaceae *in pigs and variations in virulence support the theory of genetic diversity [15,21,34,38,39]. Intestinal *C. suis *infections seem to be common in commercial pigs and most, if not all, are believed to be subclinical [12,15,17,34,72]. However, Evans postulates have been fulfilled for this pathogen [16-18], thus it is probably more harmful as some might believe. *Chlamydia suis *was involved in return to oestrus (early embryonic dead in more than 50% of the pregnant sows) in sows and inferior semen quality (decrease of sperm cell motility and dead of more than 50% of the sperm cells) in boars, as demonstrated on fattening pig farms in Belgium, Cyprus, Estonia, Germany, Israel and Switzerland [14,21,33,36,39,54]. Becker et al. [38] found a high prevalence of *C. suis *in eyes of German (90%) and Swiss (79%) ocular symptomatic pigs. In general, intensive kept pigs seemed to be pre-disposed to ocular chlamydial infection associated with conjunctivitis. Schautteet et al. [36] observed the same, examining conjunctivitis and reproductive failure on a large Estonian pig production plant.

*Chlamydia suis*, *C. pecorum *and *C. abortus *have been detected in aborted foetuses [11,13]. Eggemann et al., [21] found a significant correlation between chlamydial PCR positivity and the incidence of abortion and litters with stillborn piglets and piglets with low viability. Seropositive farms had statistically less weaned piglets per sow and litter. Hoelzle et al., [39] used two PCRs targeting the *ompA *(encoding the MOMP; 40 kDa) or *ompB *(encoding the cy**s**teine rich outer membrane protein; 60 kDa) gene, respectively. The PCR was not species-specific but was able to detect *C. suis, C. pecorum *as well as *C. abortus *in lungs, intestines and endocervical swabs of pigs. PCR amplicons were generated from 49 and 60% of pigs with respiratory or reproductive disorders, respectively. Chlamydial DNA was present in 24.5% of respiratory healthy controls and in none of the endocervical swabs from fertile control sows. They found a high prevalence of mixed *C. abortus *and *C. suis *infections in lungs and intestines using RFLP and DNA sequence analysis of *ompA*.

## 6. Public health significance

The zoonotic potential of *C. abortus *and *C. psittaci *is well documented [73]. However, to our knowledge, transmission of these pathogens from pigs to humans has not been reported. *Chlamydia suis*, previously referred to as *C. trachomatis*, might be a potential zoonotic pathogen. So far, no reports on zoonotic transmission were published.

## 7. Prevention and treatment

*Chlamydiaceae *are highly susceptible to chemicals that affect their lipid content or the integrity of their cell walls. Cleaning of equipment and stables of infected pigs is important because *Chlamydiaceae *can survive for up to 30 days in faeces and bed materials. Disinfection with most common detergents and disinfectants will inactivate *Chlamydiaceae*. The following disinfectants can be used to inactivate the organism: 1:1000 dilution of quaternary ammonium compounds, 70% isopropyl alcohol, 1% Lysol, 1:100 dilution of household bleach or chlorophenols [74,75]. Common infection sources, infection routes, possible vectors and infection kinetics on the farm have not been examined.

Current infections are being treated by means of antibiotics. Generally, tetracyclines (chlortetracycline, oxytetracycline, doxycycline) are the drugs of choice to control the disease because they are most effective. Quinolones (enrofloxacin) or macrolides (erythromycin) can be administered, in case of an infection with a tetracycline resistant *C. suis *strain. Enrofloxacin might present a solution in case of tetracycline resistant *C. suis *strains [33].

Pollman et al. [76], demonstrated the beneficial effect of a probiotic strain of *Enterococcus faecium *(NCIMB 10415) on reducing carryover infections from naturally *Chlamydiaceae *infected sows to newborn piglets. The probiotic strain is licensed by the European Union as a feed supplement for animals.

So far, no vaccines are available. Chlamydial vaccines produced for *Chlamydiaceae *in other animals would likely have no efficacy in pigs, as the strains are both genetically and serologically very different. Therefore, Knitz et al. [77] used a herd-specific, formalin-inactivated *C. abortus *strain (OCHL03/99) originating from vaginal discharge of sows to study the humoral immune response in immunized breeding sows, as a preliminary study towards vaccine development. Compared to the control group, vaccinated animals showed a marked primary and secondary IgG serum antibody response. Protection was not examined. Recently, Zhang et al. [78] presented data on vaccination of mice using a porcine *C. abortus *strain. They obtained a protective immune response following co-vaccination using a DNA vaccine together with recombinant MOMP.

Nevertheless, it is obvious that we need more progress in understanding protective and (possible) pathological immune mechanisms in pigs, before a potential vaccine candidate for *Chlamydiaceae *can be generated.

## 8. Authors' contributions

KS has compiled the literature concerning the subject and has written the manuscript. DV has contributed by carefully correcting the manuscript. All authors read and approved the final manuscript.

## 9. Competing interests

The authors declare that they have no competing interests.
